# Molecular Mechanisms of Juvenile Nasopharyngeal Angiofibroma: A Narrative Review

**DOI:** 10.3390/curroncol33030147

**Published:** 2026-03-03

**Authors:** Xingchen Liu, Junying Hu, Weigang Gan, Feng Liu, Bing Zhong

**Affiliations:** Department of Otolaryngology Head and Neck Surgery, West China Hospital of Sichuan University, 37 Guoxue Lane, Chengdu 610041, China; lxc4181@gmail.com (X.L.); hujunying2001@163.com (J.H.); coolor123@163.com (W.G.)

**Keywords:** juvenile nasopharyngeal angiofibroma, molecular markers, targeted therapy

## Abstract

Juvenile nasopharyngeal angiofibroma (JNA), a rare vascular tumor in adolescent males, involves dysregulated angiogenesis and hormonal interplay. Key molecular drivers include HIF-1α, VEGF, bFGF, and β-catenin, promoting tumor growth via pathways like Wnt/β-catenin and Ras signaling. Androgens and estrogen modulate progression, though mechanisms remain debated. Targeted therapies reduce tumor proliferation and vascularity in preclinical studies, yet clinical translation is hindered by drug resistance and inconsistent biomarker expression. Hormonal and MMP-targeted approaches also show potential but require validation. This review consolidates JNA’s molecular landscape, emphasizing the need for personalized strategies, biomarker refinement, and combination therapies to improve therapeutic outcomes for this challenging tumor.

## Simple Summary

JNA is a rare, benign but highly vascular tumor that mostly affects teenage boys. Surgery cures most patients, yet heavy bleeding during operations and tumor regrowth remain major problems. This review brings together what is known about the biological signals that drive JNA, including pathways that promote new blood vessel formation, cell growth, and hormone-related regulation that may explain its age and sex patterns. We outline how these pathway hubs could help prioritize practical biomarkers and support the development of targeted medicines, either to reduce blood supply before surgery or to treat stubborn or recurrent disease.

## 1. Introduction

**Figure 1:**
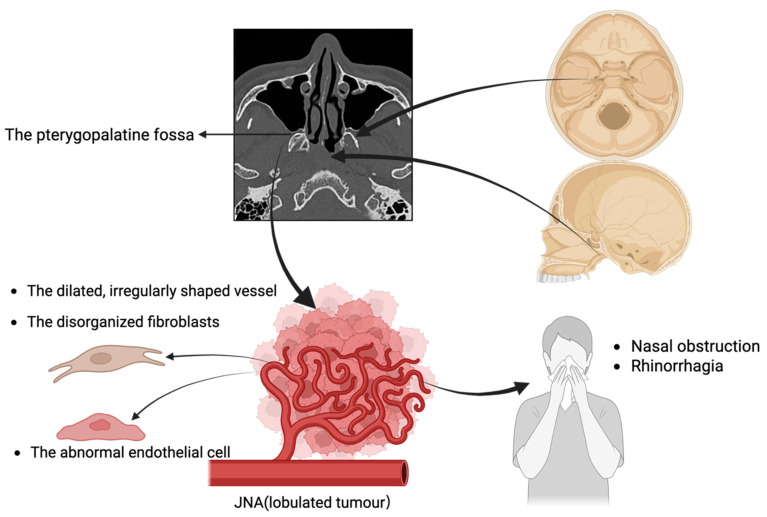
Clinical characteristics of JNA. JNA is commonly found in the pterygopalatine fossa, and the tumor typically has a lobulated appearance, usually causing nasal obstruction and rhinorrhagia.

**Figure 2:**
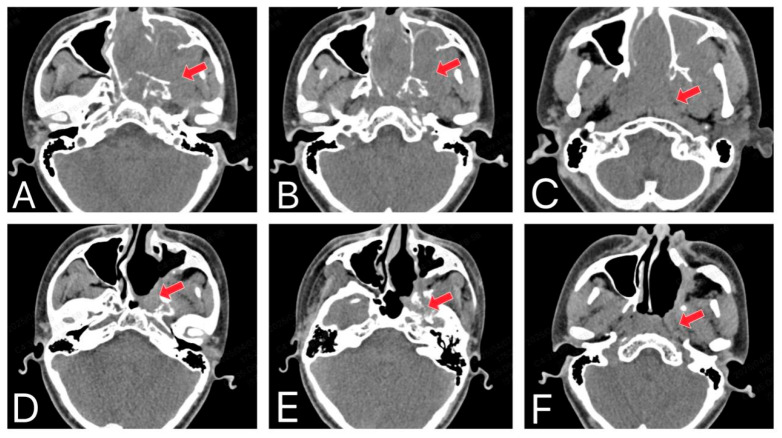
The invasive nature and recurrence of JNA. (**A**–**C**) CT shows that the original JNA originates from the posterolateral wall of the nasal cavity, near the pterygopalatine fossa region, and invades the surrounding bone. (**D**–**F**) The CT shows a recurrent JNA after surgery (The red arrow in the image indicates the lesion site).

Vascular tumors of the nasal area are diseases characterized by abnormal proliferation or tumor formation of blood vessels. While vascular tumors are relatively common in the head and neck region, they are rare in the nasopharynx and nasal cavity [1]. The most common nasopharyngeal tumor is juvenile nasopharyngeal angiofibroma (JNA), often seen in young males, known for its vascularity and bleeding risk, originating from the nasal cavity’s posterior lateral wall near the pterygopalatine fossa [2] (Figure 1). This benign tumor can exhibit locally destructive behavior [3]. The most common symptoms include nasal obstruction and rhinorrhagia [4]. JNA vasculature exhibits dilated, irregularly-shaped vessels with thin walls and abnormal endothelial cells. The tumor stroma contains hyperplastic, disorganized fibroblasts [5]. Sparse smooth muscle cells impair vascular contraction, contributing to uncontrollable bleeding in JNA [6] (Figure 2). Meanwhile, vascular fibromas of the head and neck typically occur in the nasopharynx but can also arise in other locations, particularly within the sinuses such as the maxillary sinus [7].

**Figure 3:**
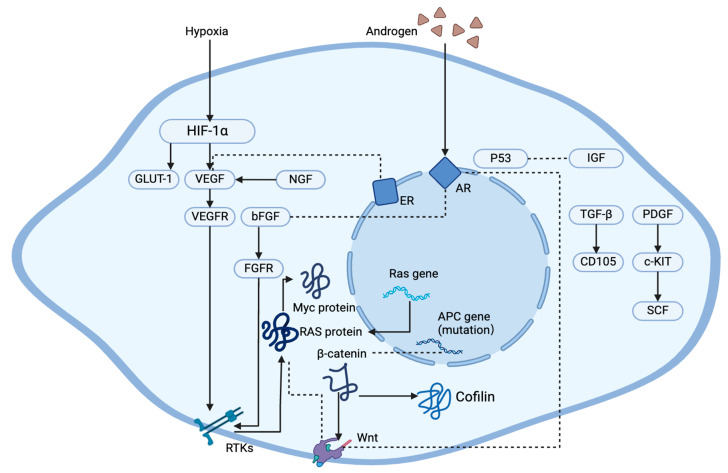
Key molecular pathways and main factors implicated in the pathogenesis of JNA. Key molecular and genetic components involved in tumorigenesis and cellular signaling pathways. Interactions among components drive angiogenesis, cell proliferation, local invasion, and postoperative recurrence, indicating therapeutic targets (solid lines indicate direct relationships, while dashed lines show indirect interactions or cooperative modulation).

JNA is increasingly recognized and frequently highlighted in case reports and molecular protein studies, yet its molecular mechanisms remain unclear. This article reviews the established molecular mechanisms of nasopharyngeal angiofibroma, focusing specifically on JNA, to aid in developing future treatment strategies (Figure 3).

Extranasopharyngeal angiofibroma (ENA) is best viewed as a related but clinically distinct subgroup, spanning a broader age range and diverse primary sites [8]. Compared with classic JNA, ENA may show less stereotyped hypervascular imaging features, which can influence decisions on angiography and preoperative embolizations [9]. Given that ENA evidence is largely case-based, and biomarker reporting is mainly diagnostic rather than pathway-oriented, molecular inferences from classical JNA should be extrapolated to ENA with caution [10,11].

## 2. Proteins Associated with JNA Pathologic Progression

### 2.1. Angiogenic Signaling

#### 2.1.1. The Effect of VEGF in JNA

HIF-1α shows a strong correlation with VEGF [12]. It accumulates in endothelial cells under hypoxic conditions, binds to the VEGF gene promoter, and induces VEGF gene expression. Concurrently, hypoxia stabilizes HIF-1α, which serves as a primary stimulus for tumor cells to increase VEGF production [13]. VEGF receptors are categorized into three types, with VEGFR-1 and VEGFR-2 playing significant roles in tumor-associated vasculature, as well as in physiological angiogenesis. VEGFR-1 often dampens VEGF-A signaling by sequestering the ligand on endothelial cells, but it can convey inflammatory cues in VEGF-B/PlGF-enriched stromal or myeloid contexts [14,15], whereas VEGFR-2 is the principal driver of angiogenesis; VEGFR-2 mediates most downstream angiogenic effects of VEGFA, including microvascular permeability, cell proliferation, migration, and survival [16,17]. Numerous studies indicate high expression of VEGF in JNA. Zhang noted that the expression levels in JNA endothelial cells were significantly higher than those in orbital cavernous hemangiomas [16]. Mishra considered VEGF to be the most extensively studied molecular marker in JNA, suggesting that its higher expression may increase vascular density rather than reflect aggressive tumor growth [18]. Brieger’s research indicates strong VEGF expression in JNA’s vascular endothelium and lower in tumor stroma, suggesting it may cause high vascular density without large or aggressive tumors [19]. Anupam Mishra compared JNA tumor tissue with polyp tissue and confirmed significant VEGF expression in hemangiomas [20,21], noting that higher VEGF levels correlate with increased bleeding and a higher rate of recurrence [22]. Conversely, Zhang’s research on JNA found no significant relationship between high VEGF expression and patient recurrence rates, suggesting that high levels of VEGF are mainly present in stromal cells [23]. Ansari Nagar noted that while VEGF is found in tumor stromal cells, it is primarily localized in endothelial cells [24]. Despite the expected high expression of VEGF, a study by Ngan on seven preoperative embolized JNA samples failed to detect VEGF in endothelial cells, attributing this phenomenon to the embolization process [25].

Studies on VEGF expression in JNA—specifically whether it is located in endothelial or stromal cells—and its link to clinical outcomes like bleeding and recurrence have shown inconsistent results. This indicates that VEGF likely does not act alone; instead, its function may be coordinated or counterbalanced by other factors in the tumor microenvironment, such as bFGF and PDGF [21]. Moreover, hypoxia caused during the collection of surgical resection specimens can compromise the accuracy of VEGF-related biomarkers [26]. Therefore, using VEGF expression levels alone as a biomarker or treatment target should be approached with caution.

Divergent VEGF/VEGFR findings in JNA largely reflect embolization-related effects, intra-tumoral heterogeneity, compartment-specific scoring, non-standardized IHC/MVD methods, and small heterogeneous cohorts. Accordingly, VEGF-related data require standardized clinicopathologic annotation and harmonized staining and scoring (Table 1) [18,25]. Overall, VEGF expression appears to indicate vascular density and perioperative bleeding risk rather than intrinsic tumor aggressiveness and—given its strong methodological and clinical sensitivity—should not be used alone to predict recurrence or guide therapy.

#### 2.1.2. The Effect of bFGF in JNA

bFGF is a growth factor that promotes cell proliferation and angiogenesis. bFGF activates intracellular signaling pathways by binding to receptors on the cell surface, thereby affecting cell growth and differentiation [27]. Numerous studies have demonstrated high expression of bFGF in JNA. Thomas confirmed this using RNA sequencing (RNA-seq) and provided relevant data on VEGF and bFGF [28]. Schuon also supported this conclusion in a retrospective, descriptive, multicenter study, suggesting that overexpression of bFGF may lead to increased angiogenesis and tumor cell proliferation [29]. In Pandey’s study, bFGF was considered the second most potent angiogenic factor in the studied JNA population (following VEGFA) [20]. Safhi found that smokers among JNA patients had higher recurrence rates and bleeding intensity, highlighting the potential role of FGFR3 as a key molecular factor in JNA [30]. Although the relationship between FGF and JNA is relatively clear, Mishra suggested tumor volume inversely correlates with FGF expression, where higher FGF relates to smaller tumors, more bleeding, and recurrence. Mishra observed increased bFGF but unchanged AR expression, providing no support for AR-mediated androgen dependence [18].

bFGF findings differ because early studies emphasized angiogenic activity, whereas later work highlighted compartment-resolved expression, with higher stromal levels in highly vascularized tumors [29,31]. Sampling bias and methodological variability further influence whether bFGF appears endothelial- or stroma-dominant (Table 1).

#### 2.1.3. The Effect of PDGF in JNA

PDGF promotes mitosis in capillary endothelial cells and stimulates the synthesis of the extracellular matrix [32]. Roach observed that PDGF levels increased during the proliferation phase when studying infantile hemangiomas [33]. The research found that PDGF signaling serves as an intrinsic negative regulator of hemangioma regression. Currently, enhanced PDGF signaling is associated with vascular diseases and fibrosis [34], suggesting its potential role in the neovascularization and fibrosis of JNA. However, in current studies on the JNA population, Mishra posits that PDGF appears to have a lesser angiogenic effect compared to FGF and VEGF [18].

Evidence for PDGF involvement in JNA is limited and heterogeneous, as most data derive from mRNA-level associations rather than direct protein localization or functional studies [35]. Consequently, PDGF is generally viewed as a potentially relevant but low-evidence pathway pending further validation [20].

#### 2.1.4. The Effect of NGF in JNA

NGF is a member of the growth factor family and also belongs to the neurotrophic factor family, playing a regulatory role in angiogenesis [36]. NGF is associated with various growth factors and can directly increase the expression of VEGF in endothelial cells [37]. Zhang found NGF in fibroblasts and endothelial cells of JNA, indicating that fibroblasts may secrete NGF, promoting vascular growth in tumors [38]. However, research on NGF in hemangiomas remains limited, which could be a direction for our future studies. Various types of growth factors interact with each other. Schuon found high bFGF, TGF-β1, and VEGFR-2 levels linked to increased vascular density in JNA [39]. Mishra also studied and confirmed interactions between growth factors and protein-coding genes in JNA in another article [21].

#### 2.1.5. The Effect of TGF-β1 in JNA

TGF-β1 can activate the proliferation of fibroblasts and induce angiogenesis [40]. However, the expression of TGF-β1 in JNA remains controversial. Studies showed positive TGF-β staining [40,41]. However, in recurrent cases, only three were stained for TGF-β. Nagai confirmed TGF-β1 in JNA lesions, suggesting its role in tumor fibrous development [35]. In contrast, Zhang compared TGF-β3 expression in JNA and nasal polyps, finding no significant differential expression of TGF-β3 between the two [38]. Endoglin (CD105) is a key endothelial cell co-receptor of the TGF-β superfamily [42]. CD105 is currently used to assess microvessel density (MVD) [43], a useful prognostic marker for poor outcomes following radical resection in JNA patients. Wang’s study demonstrated that CD105 is associated with JNA recurrence [44].

Discrepant findings regarding TGF-β signaling in JNA likely reflect target mismatch and methodological heterogeneity rather than true biological contradiction. Current evidence supports the role for TGF-β in stromal remodeling, but not as a consistent prognostic or therapeutic marker. Discrepant TGF-β findings in JNA largely reflect target mismatch such as TGF-β3 [38,39,40], compartment-specific assessment, and methodological heterogeneity across small, clinically variable cohorts (Table 1) [29,41]. Overall, current evidence links TGF-β signaling primarily to stromal remodeling rather than intrinsic tumor behavior and does not support its use as a reliable prognostic marker or standalone therapeutic target.

#### 2.1.6. The Effect of Transmembrane Protein in JNA

CD34 is a transmembrane protein marking endothelial cells, hematopoietic progenitor cells, and endothelial progenitor cells, and is a diagnostic marker for vascular tumors, acting as an endothelial marker in angiogenesis [45]. Immunoexpression of CD34 is significantly higher in angiofibromas compared to cavernous hemangiomas [16], and these data support the notion that JNA exhibits the biological characteristics of a tumor arising from angiogenic tissues. In recent years, there has been extensive research on the relationship between CD34 and JNA. Studies assessing MVD in JNA, through CD34 immunostaining have found that MVD is significantly correlated with tumor volume in JNA, and higher MVD may serve as a risk factor for recurrence [46,47].

### 2.2. Hormones and Receptors

Research on sex hormones and hemangiomas has long been a focal point for experts and has been accompanied by certain controversies. JNA primarily occurs in young males, and the prevailing view today is that it is mainly regulated by androgens [48]. Kumari conducted a comprehensive analysis of JNA using whole exome sequencing provided the first evidence that genes on the Y chromosome are involved in JNA [49]. Androgens and growth factors are linked, with studies showing that high PDGF levels in adolescents may boost their synergistic effect [50]. Häggström reported that testosterone stimulates the proliferation of endothelial cells and angiogenesis in the rat prostate, a process that may be mediated by testosterone-induced VEGF synthesis [51]. Mishra confirmed the role of elevated androgen levels (mediated by VEGF upregulation) in angiogenesis within JNA [18].

Currently, some scholars are investigating the relationship between estrogen and JNA. Montag reported consistent ER-β expression in JNA, while ER-α and PR were not detected, highlighting the importance of isoform-specific assessment when interpreting ER findings [52]. However, Wang demonstrated high expression of estrogen receptors in JNA specimens [53]. Schick studied sex hormone receptors in juvenile vascular fibromas, finding high expression of estrogen receptor alpha, FSHR, and LHR in JNA [39]. Researchers found LHCGR hormone receptor crucial for sex differences, puberty, and JNA progression, with LHCGR+ cells near blood vessels indicating LH/hCG signaling roles in JNA’s vascularization and growth [54]. However, the measurement of estrogen receptors relies on a monoclonal antibody targeting estrogen receptor alpha, which may fail to detect estrogen receptor beta, thus explaining the reported discrepancies in estrogen receptor expression [55].

Inconsistent ER findings in JNA largely reflect incomplete subtype coverage, with early studies focusing on ERα alone and later analyses demonstrating predominant ERβ expression with only occasional ERα positivity, compounded by variability in compartmental localization and clinical context (Table 1) [55,56,57]. Overall, available evidence supports ERβ-dominant signaling in JNA; however, receptor expression alone does not establish hormone dependency and should not be overinterpreted as a therapeutic indication.

High VEGF levels correlate with estrogen receptor dominance; estrogen inhibits JNA formation but facilitates VEGF signaling [58]. Massimo Ralli found that post-menopausal declines in estrogen levels could promote the proliferation of JNA cells in a case involving an elderly female patient [52]; while a few case-based observations have suggested potential changes in tumor behavior during pregnancy, current evidence is insufficient to support a consistent or generalizable effect on JNA progression [59]. Nevertheless, some studies hold opposing views, suggesting that the role of hormones in JNA remains to be further investigated. Saylam assessed proliferation, angiogenesis, and hormonal markers in JNA, concluding that ER and PR are not significant in its pathogenesis despite some positives [41]. Based on in-depth studies of sex hormones, the pathogenesis of JNA likely involves estrogen and androgens, needing further investigation.

### 2.3. Cell Proliferation, Survival, and Signal Transduction

#### 2.3.1. The Effect of β-Catenin in JNA

β-Catenin promotes calcium-dependent cell adhesion via cadherins, forming a crucial cadherin–catenin complex for cell adhesion; its dysfunction can lead to tumor progression [60]. c-KIT can induce tumor malignancy through the Wnt/β-catenin pathway, regulated by the APC gene [61]. The inhibition of this pathway alleviates pulmonary fibrosis, and desmoid tumors arise from Wnt/APC/β-catenin alterations [62].

A connection between JNA and familial adenomatous polyposis (FAP) has been established. Reports shows that the diagnosis frequency of JNAs in FAP patients is 25 times higher than that in age-matched populations [63]. However, Klockars, in a retrospective study of JNA, concluded that there is no significant correlation, a view shared by Mishra [64,65]. The contradictory findings likely stem from differences in study populations. Research supporting an association typically studies confirmed FAP patients with APC mutations, who show a higher JNA incidence. Studies finding no link often examine sporadic JNA cases in smaller cohorts, making rare associations hard to detect. Mechanistically, both sporadic JNA and FAP-related JNA converge on Wnt/β-catenin pathway activation, explaining their similar phenotypes. While β-catenin activation is a key mechanism in JNA, its epidemiological link to FAP needs more evidence. Clinically, young JNA patients should still be assessed for FAP, especially if symptoms or family history are present.

Ponti studied JNA and APC mutations associated with FAP syndrome and found that all sporadic and familial JNA tumors showed nuclear staining for β-catenin [66]. Abraham’s analysis of sporadic JNA in non-FAP patients found somatic β-catenin and APC mutations, showing nuclear β-catenin accumulation in stromal cells, indicating they are the tumor cells [67]. He also discovered that sporadic JNAs had a higher frequency of β-catenin mutations, and the nuclear expression of β-catenin in sporadic JNA stromal cells was higher compared to nasal polyp stromal cells [38]. In contrast, Rippel found strong β-catenin in JNA stromal and endothelial cells but argued it does not conclusively support stromal cells as tumor cells [68]. Additionally, research has shown that β-catenin is associated with androgen receptors [69], leading Rippel to suggest that the increased β-catenin expression in angiofibroma is related to the typical growth stimulation of this tumor in pubescent males.

#### 2.3.2. The Effect of Ras Proteins in JNA

Ras proteins are located downstream of growth factor signaling pathways. When growth factors bind to receptor tyrosine kinases (RTKs) on the cell surface, they subsequently activate Ras proteins [70]. The Ras family includes HRAS, KRAS, and NRAS, with their abnormal activation linked to cancer development [71]. Coutinho’s study first demonstrated that Ki-Ras and Ha-Ras gene mutations are not associated with the development of JNA [72]. However, recent research by Mishra confirmed that enhanced Ras signaling is linked to post-pubertal onset, smaller tumors, skull base involvement, and recurrence [22], highlighting the VEGF/FGF/Ras complex’s critical role in JNA. Furthermore, Mishra’s study demonstrated a significant interaction between FGF, VEGF, and Ras [21].

#### 2.3.3. The Effect of c-KIT in JNA

c-KIT (CD117) is a tyrosine kinase receptor that belongs to the platelet-derived growth factor receptor (PDGFR) family [73], and it is believed to play a critical role in tumor pathogenesis [74]. Renkonen conducted a retrospective analysis of clinical pathological data from JNA patients, discovering c-KIT expression in both stromal and endothelial cells [75]. Furthermore, there was a strong correlation between c-KIT expression in endothelial cells and cell density. Zhang confirmed that c-KIT immunoexpression in the cytoplasm of stromal cells from JNA was higher than that in polyp stromal cells, occurring at a higher frequency [38]. Renkonen found a correlation between c-KIT immune expression and tumor cell density, indicating that vascular components may aid tumor growth [75]. Higher c-KIT levels were associated with adolescence, rapid growth, skull base involvement, and recurrence [22]. However, Pauli analyzed various growth factors and oncogenes in 54 cases of nasopharyngeal angiofibromas, surprisingly finding that c-KIT was negative in all cases [76]. Meanwhile, its ligand stem cell factor (SCF) plays an important role in the function of sex hormones, indicating a possible relationship between c-KIT and hormones, though the details remain unclear [77].

#### 2.3.4. The Effect of Myc in JNA

Myc protein is a transcription factor that regulates the expression of various genes, thereby influencing cell growth and proliferation [78]. Myc protein is located downstream of Ras protein, as the activation of Ras can enhance the expression of Myc through the RAF/MEK/ERK signaling pathway [79]. Renkonen detected C-Myc expression exclusively in JNA stromal cells [75]. Mishra found that Myc mRNA expression was significantly increased in different regions of JNA molecular expression and did not parallel with the allergic rhinitis [18]. Meanwhile, Amaya detected Myc levels in tissue samples from different benign and malignant vascular tumors, showing widespread expression of Myc [80]. Consequently, Mishra argues that C-Myc alone cannot be considered a therapeutic molecular target, as its overexpression or underexpression can lead to an aggressive phenotype, depending on the pathways involved [22].

#### 2.3.5. The Effect of IGF in JNA

IGFs, particularly IGF-1 and IGF-2, are linked to hemangioma diseases; IGF-1 boosts HemSC proliferation and adipocyte differentiation, while IGF-2 is found in tumor vasculature [81]. However, Zhang’s study utilizing standard immunohistochemical techniques on JNA did not detect IGF-1R immunoreactivity in the specimens [38]. Elevated levels of IGF-II suggest a higher risk of cancer, although IGF2R is generally considered a tumor suppressor. Nagai’s research indicates that IGF-II may have a synergistic effect with p53 in JNA, suggesting IGF-II as a potential growth regulator for this condition [35].

### 2.4. Extracellular Matrix Remodeling and Migration

#### 2.4.1. The Effect of Proteolytic Enzyme in JNA

Matrix metalloproteinases (MMPs), particularly MMP-2, MMP-9, and MT1-MMP, are linked to angiogenesis by degrading ECM, releasing angiogenic factors, generating inhibitors, and revealing key ECM sites [82]. MMP-9 correlates with vascular wall instability and hemorrhagic brain diseases [83]. Duerr identified MMPs with gelatinase/collagenase activity in JNA, indicating a disrupted balance with TIMPs and increased MMP activity [84]. MMP-9 was found in poorly differentiated and stromal cells of JNA patients, but not in mature endothelial cells. High MMP-9 expression is associated with a significantly higher recurrence rate in JNA patients, indicating that it may be a poor prognostic factor for those undergoing surgery [85].

#### 2.4.2. The Effect of Cofilin in JNA

The Wnt/β-catenin pathway contributes to the maintenance of endothelial cell integrity by regulating actin dynamics under homeostatic conditions, with its downstream signaling converging at Rac1 [86]. Rac1 controls the dynamics of the subcellular actin filament severing protein, cofilin [87]. A study found cofilin in irregular smooth muscle cells, pericytes, poorly differentiated stromal cells, and plump cells, but not in inactive fibroblasts or mature endothelial cells. High cofilin expression in JNA patients correlated with a higher recurrence rate [88].

#### 2.4.3. The Effect of Ntigen in JNA

Ntigen (NG2) is a type 1 transmembrane protein expressed in pericytes, progenitor cells, and glioblastoma cells. Protein kinase CK2 regulates endothelial cell functions [89] and NG2-dependent signaling in pericytes and GBM [90]. Boewe’s study found that JNA expresses proteoglycan NG2, influencing cancer cell migration and that CK2 activity loss inhibits JNA cell proliferation and migration [91]. Currently, research on neural glial antigen remains limited, and it is hoped that more studies will be available in the future for further reference.

### 2.5. Glucose Metabolism and Hypoxic Response

#### 2.5.1. The Effect of HIF-1α in JNA

HIFs are transcriptional activators that regulate genes associated with hypoxia [12]. Hypoxia accelerates tumor cell growth, with HIF boosting proliferation and invasive behavior, increasing VEGF and TGF expression, and promoting angiogenesis [92]. There are three HIF subtypes: HIF-1, HIF-2, and HIF-3 [93]. HIF-1 and HIF-2 have been extensively studied in the context of tumor immune evasion. Strong expression of HIF in hemangiomas has been confirmed; in studies related to JNA, Song reviewed 70 cases in 2007 and found higher expression of HIF-1α in younger or recurrent patients’ tumor cells [23]. This finding indirectly supports the notion that younger JNA patients are more likely to experience recurrence [23,94], contradicting Liu’s assertion that age is unrelated to recurrence rates [6]. HIF-1α expression varies with embolization status and sampling location, as hypoxia signaling may persist in viable tumor regions after embolization [25]. Compartment-specific analyses show enrichment in highly vascularized areas and context-dependent associations with recurrence or younger age [23,29].

#### 2.5.2. The Effect of GLUT-1 in JNA

Glucose transporter type 1 (GLUT-1) is an erythrocyte-type glucose transporter protein and a downstream target of HIF-1α, playing a critical role in cellular responses to hypoxia [95]. Its high expression in the endothelial cells of juvenile hemangiomas has led to the consideration of GLUT-1 as a specific marker for distinguishing hemangiomas from other benign vascular lesions, where GLUT-1 is typically negative [96]. In a retrospective case series of adult periocular LCH, GLUT-1 was found to be negative in tumor-associated endothelial cells [97]. The expression of GLUT-1 in JNA appears to be contentious. Nonogaki reported JNA and vascular malformations as GLUT-1 negative, while hemangiomas were positive [98]. Endothelial markers indicate JNA vasculature’s hyperproliferative state [19]. Subsequently, Renkonen re-evaluated 27 cases, finding positive GLUT-1 expression in some JNA specimens, with endothelial GLUT-1 correlating to higher tumor staging, suggesting JNA is not a vascular malformation [99].

GLUT-1 expression in JNA remains controversial. This variability may arise from several factors, such as differing staining thresholds, variations in sample processing, and the tumor’s inherent heterogeneity [100]. Additionally, since GLUT-1 is a hypoxia-responsive protein, its expression can be influenced by local hypoxia within the tumor [101], as well as by vascular maturity, endothelial differentiation, underlying etiology [102], and patient age [103]. GLUT-1 expression in JNA is context-dependent and strongly influenced by hypoxia and endothelial differentiation status. While its positivity in selected cases argues against a pure vascular malformation phenotype, GLUT-1 currently lacks sufficient robustness to serve as a diagnostic or prognostic discriminator in JNA.

**Table 1:** Discrepant expression patterns of representative molecular markers in juvenile nasopharyngeal angiofibroma and plausible sources of heterogeneity.

Marker	Main Discrepant Findings	Plausible Sources of Heterogeneity
**VEGF**	Positive [18,19,22] (stromal cells [23], endothelial cells [24]) Negative (endothelial VEGF [25])	Embolization [25]. Non-harmonized IHC and MVD methods. Small mixed cohorts [18].
**TGF-β (β1/β3)**	Positive (TGF-β1 [35,40,41], TGF-β3 [38])	Target mismatch [38,40]. IHC workflow variability [41]. Embolization/hypoxia. Inadequate stratification.
**GLUT-1**	Negative [98] Positive [99]	Threshold/interpretation. Hot-spot and compartment bias [99]. Embolization/hypoxia timing. Fixation/retrieval [99]. Cohort composition.
**ER (ERα/ERβ)**	Negative [56] Positive (ERβ [55], ERα [57]).	Isoform coverage [55,56,57]. IHC workflow [55]. Compartment. Hormonal context.

## 3. Potential Targeted Therapy

Table 2 provides a summary of the potential relevant treatment options discussed in the article.

### 3.1. Potential of HIF Inhibitors in JNA Treatment

Based on the aforementioned research, several potential targeted therapies for JNA have emerged. Currently, around 40 HIF inhibitors are under clinical investigation [104]. Belzutifan is a new HIF-α inhibitor for hemangioblastoma [105], while propranolol targets the HIF-1α-VEGF-A pathway to inhibit hemangiomas [106]. The principle behind these therapies is that inhibiting HIF, and thus angiogenesis, may help reduce tumor growth and the recurrence of JNA. HIF inhibitors are widely used for malignant tumors, but JNA is typically benign but locally aggressive, so the risk–benefit profile of anti-HIF therapy requires careful evaluation [107]. In the field of oncology, further clinical studies are needed to evaluate HIF-targeted therapy in JNA.

### 3.2. Advances and Limitations of Growth Factor Targeted Therapy

Targeted therapy against VEGF has been partially applied in the treatment of vascular tumors [108,109] with positive outcomes. SU5416 is an early VEGFR inhibitor that has primarily served as a proof-of-concept tool in angiogenesis research, with limited translational relevance at present [100]. Research has suggested that SU5416 may have potential for tumor treatment [110]. Thomas’s study found that SU5416 inhibits the proliferation of JNA cell lines and reduces angiogenesis by inhibiting the VEGF signaling pathway [111]. This indicates that SU5416 may suppress the growth and local extension of JNA. However, Sweeney noted that using anti-VEGF therapy directly risks misclassifying non-vascular tumors as the vascular ones, complicating diagnosis and treatment [112]. Additionally, Park concluded that therapies targeting the VEGF–VEGFR pathway are ineffective for most patients with complex vasogenic diseases [113].

AZD4547 is a highly efficient pan-inhibitor of FGFR tyrosine kinases. Recently, Thomas investigated the effects of AZD4547 on JNA fibroblasts and found that it effectively reduces the proliferation of JNA fibroblasts [111]. As the concentration of AZD4547 increases, the survival rate of JNA fibroblasts significantly decreases.

SU5402 is an FGFR inhibitor that blocks bFGF binding and has been studied for its role in tumor growth and angiogenesis, but its use in nasopharyngeal angiofibroma is unexplored [114].

Imatinib, a PDGFR family-specific tyrosine kinase inhibitor, has therapeutic effects on tumors that express abnormal forms or high levels of corresponding target proteins [115]. High levels of c-KIT expression in vascular tumors could serve as a target for imatinib therapy. A study found that normal tissues and tumors show positive c-KIT staining unrelated to Kit or PDGFRB mutations, leading Pauli to suggest imatinib mesylate is unlikely to help without these mutations [76]. Currently, the relationship between c-KIT protein and KIT gene or PDGFRB in JNA has not been clearly established, thus warranting further investigation.

MiR-139-5p is a microRNA (miRNA) that can influence the IGF-1/IGF-1R pathway by regulating IGF-1R expression, thereby affecting the proliferation and migration of hemangioma stem cells (HemSCs) [116]. However, because IGF-1 shows poor immunoreactivity in JNA, miR-139-5p has not yet been applied in this context [38]. Nonetheless, Ou reported that IGF2R knockdown inhibited vascular tumor cell proliferation and induced apoptosis, suggesting IGF2R as a potential therapeutic target in JNA [117].

Imaging and DSA remain central to diagnosis and operative planning in JNA. When definitive resection is delayed or staged, VEGF/VEGFR signaling and CD105-defined microvessel activity are most relevant to vascularity control, whereas MMP-9 has been linked to postoperative persistence [24]. Because these readouts vary with embolization-related hypoxia, sampling compartment, and IHC workflows, they are best viewed as hypothesis-generating and may mainly inform risk-aware follow-up rather than change routine management [18].

### 3.3. Other Potential Targets: β-Catenin and Cofilin

β-catenin inhibitors suppress prostate cancer cell growth by disrupting the interactions between β-catenin/T-cell factor and β-catenin/androgen receptor proteins [118]. Research on cofilin is limited, but Pan showed propranolol inhibits norepinephrine-induced cell invasion in infantile hemangiomas by reducing MMP-9, VEGF, and p-cofilin expression [119]. Such targeted drugs have not yet been utilized in JNA, making them potential new therapeutic targets for this condition.

### 3.4. Hormonal Therapies: Testosterone Blockers and Estrogen Applications

Testosterone blockers have been shown to reduce the growth rate of JNA fibroblasts [39]. Flutamide and medroxyprogesterone acetate’s effects on JNA have been studied, but flutamide’s role is controversial. It suppresses vascular fibromas’ proliferation [57]. Currently, the main purpose of using flutamide in JNA is to reduce tumor size [120]. Studies indicate this drug may cause osteoporosis in prostate cancer treatment, while Phalak’s research observed cerebrospinal fluid leakage post-medication in JNA treatment, attributing it to tumor growth and shrinkage rather than osteoporosis, needing further investigation [59].

Simultaneously, studies have explored the use of estrogen in treating JNA based on the principle of countering androgens with estrogen [121]. Tamoxifen can inhibit the proliferation of vascular fibromas [57]. It is more commonly used to manage gynecomastia, and its potential risks mainly relate to thromboembolic and metabolic adverse effects [122,123]. Doxycycline and minocycline, which are tetracycline antibiotics, can enhance vascular stability and thereby reduce the risk of spontaneous bleeding from vascular malformations by decreasing MMP-9 activity [124]. However, a study on hemangiomas indicated that patients experienced significant side effects from oral doxycycline taken 2 weeks preoperatively, leading to its exclusion from vascular tumor treatment [125].

## 4. Research Models and Integrated Systems in JNA

Most mechanistic studies still rely on primary JNA fibroblasts and endothelial cells from surgical specimens to probe growth-factor responses and pathway activity [111]. These cultures are difficult to expand, vary between batches, and cannot capture the 3D tumor–stroma–vasculature context; moreover, because specimens come from selected surgical cases, selection bias and perioperative confounding can limit comparability. More reproducible and scalable model platforms remain a clear priority.

Methodological limitations also constrain translation: the literature is largely retrospective and small, with non-standardized outcome definitions and reporting. Independent multi-center replication and orthogonal confirmation across assays are still uncommon, which weakens confidence in proposed targets and biomarkers. Addressing these gaps will require harmonized sampling and shared outcome to support reproducible validation [126,127,128].

## 5. Extending the Molecular Perspective to Extranasopharyngeal Angiofibroma

ENA warrants separate molecular-pathologic consideration because most available evidence remains case-based and is primarily centered on diagnostic immunophenotyping rather than systematic pathway profiling [129]. In ENA presenting in the anterior maxillary region, immunostains such as vimentin, CD34, and α-SMA have been repeatedly used as practical adjuncts to support the diagnosis when the morphology overlaps with other fibrovascular lesions [8]. Across ENA case reports and small series, endothelial markers, including CD34, CD31, and factor VIII-related antigen are typically interpreted as highlighting the irregular vascular component and confirming true endothelial-lined channels; in this context, they function mainly as compartment-mapping tools rather than quantitative readouts of angiogenic pathway activity [11]. Vimentin is commonly used to support a mesenchymal/fibroblastic stromal phenotype, consistent with the fibrovascular nature of angiofibroma [8]. α-SMA may label perivascular smooth muscle elements around the vessels, providing additional support for the vessel-associated component, whereas desmin negativity is frequently invoked to help exclude smooth muscle-predominant mimics in the differential diagnosis [11]. By contrast, ENA reports less consistently evaluate hormone-related and Wnt/β-catenin markers often discussed in classical JNA, and ENA-specific studies linking these immunostains to functional roles, clinical behavior, or therapeutic vulnerability remain scarce [10]. Therefore, in ENA, these immunohistochemical readouts currently serve predominantly as diagnostic and differential diagnostic markers rather than validated predictive or therapeutic biomarkers, and molecular inferences derived from classical JNA should be extrapolated to ENA with caution [9].

## 6. Conclusions

JNA is the most common type of fibroangioma in the nasopharynx; extrinsic fibroangioma is rare, but it is still a variant of JNA. The research on nasopharyngeal extrinsic fibroangioma is limited, and most of the work is published as individual case reports. It is very important to study and summarize the markers and molecular factors of these tumors. We reviewed the literature, observed and summarized the common markers of JNA, but many markers are still controversial and need to be further explored. The pathogenesis of JNA is driven by a complex interplay of genetic and molecular factors. Key genes, including HIF-1α, VEGF, and bFGF, orchestrate angiogenesis and fibroblast proliferation, while dysregulated pathways such as Wnt/β-catenin and Ras signaling promote tumor invasiveness and recurrence. Hormonal influences, particularly AR and gonadotropin receptors underpin the male predominance and adolescent onset. Additionally, c-KIT and MMPs contribute to stromal hypercellularity and vascular instability, respectively. Despite progress, controversies persist, such as the inconsistent role of TGF-β1 and limited evidence for IGF involvement. These molecular insights highlight promising therapeutic targets but underscore the need for biomarker validation and personalized approaches to overcome challenges like drug resistance and hormonal interplay. Future studies must prioritize resolving mechanistic ambiguities and translating preclinical findings into effective clinical strategies.

## Figures and Tables

**Table 2:** Potential targeted therapy in JNA. (Unless otherwise specified, the reported effects are derived from in vitro or preclinical studies. No targeted agent listed in this table has demonstrated proven clinical efficacy in juvenile nasopharyngeal angiofibroma.).

Category	Agent	Target	Revised Effects
Hypoxia signaling	HIF inhibitors	HIF-1α/HIF-2α	Preclinical evidence suggests modulation of hypoxia-driven angiogenic signaling; no clinical data are currently available to demonstrate therapeutic efficacy in JNA.
Angiogenesis	SU5416 (Semaxanib)	VEGFR-1/VEGFR-2	In vitro studies indicate reduced angiogenic signaling and proliferation; SU5416 primarily serves as an early proof-of-concept VEGFR inhibitor with limited translational relevance.
Angiogenesis Growth factor signaling	Other VEGF/VEGFR inhibitors	VEGF–VEGFR axis	Cell-based and preclinical models suggest inhibition of angiogenic pathways; clinical efficacy in JNA has not been established.
	AZD4547	FGFR1–3	In vitro inhibition of FGFR signaling reduces proliferation of primary JNA fibroblasts; these findings have not been validated in vivo or clinically.
Growth factor signaling	SU5402	FGFR	Experimental FGFR blockade demonstrates reduced proliferative activity in cell models; relevance to JNA pathophysiology remains speculative.
Growth factor signaling	Imatinib	PDGFR/c-KIT	Target engagement has been proposed based on receptor expression; functional dependency and therapeutic benefit in JNA remain unproven.
Wnt signaling	β-catenin inhibitors	Wnt/β-catenin pathway	Preclinical evidence from non-JNA models suggests pathway modulation; direct efficacy in JNA has not been demonstrated.
Hormonal therapy Hormonal therapy	Tamoxifen	Estrogen receptor modulation	In vitro studies suggest reduced stromal cell proliferation; in males, tamoxifen is clinically used to treat gynecomastia, and any application in JNA remains speculative due to potential thromboembolic risks.
	Flutamide	Androgen receptor	Limited in vitro and case-based observations suggest potential modulation of androgen-related signaling; no controlled clinical evidence supports therapeutic benefit in JNA.
Matrix remodeling	Doxycycline/Minocycline	MMP-9 inhibition	Cell-based and preclinical studies indicate reduced matrix-degrading activity and vascular instability; clinical efficacy in JNA has not been evaluated.

## Data Availability

No new data were created or analyzed in this study.

## Abbreviations

JNA: juvenile nasopharyngeal angiofibroma, HIF-1α: hypoxia-inducible factor 1-alpha; GLUT-1: glucose transporter type 1, VEGF: vascular endothelial growth factor, VEGFR: vascular endothelial growth factor receptor, bFGF: basic fibroblast growth factor, PDGF: platelet-derived growth factor, PDGFR: platelet-derived growth factor receptor, TGF-β1: transforming growth factor beta 1, IGF: insulin-like growth factor, IGF-1R: insulin-like growth factor 1 receptor, NGF: nerve growth factor, β-Catenin: beta-catenin, MMP: matrix metalloproteinase, NG2: neuron-glial antigen 2, APC: adenomatous polyposis coli, FAP: familial adenomatous polyposis, ER: estrogen receptor, PR: progesterone receptor, AR: androgen receptor, LHR: luteinizing hormone receptor, FSHR: follicle-stimulating hormone receptor, MVD: microvessel density, TIMP: tissue inhibitor of metalloproteinase, CK2: casein kinase 2.
